# First person – Anna Lennon

**DOI:** 10.1242/bio.062492

**Published:** 2026-02-03

## Abstract

First Person is a series of interviews with the first authors of a selection of papers published in Biology Open, helping researchers promote themselves alongside their papers. Anna Lennon is first author on ‘ Investigating the potential neurotoxic effects of cell-free factors by *Batrachochytrium dendrobatidis* on locomotion in *Xenopus laevis*’, published in BiO. Anna conducted the research described in this article while an undergraduate researcher in Dr Chase Kinsey's lab at Belmont University, Nashville, USA. She is now a PhD student at Indiana University in the lab of Dr Jay T. Lennon, investigating the evolution of division of the labour system engaged in dormancy.


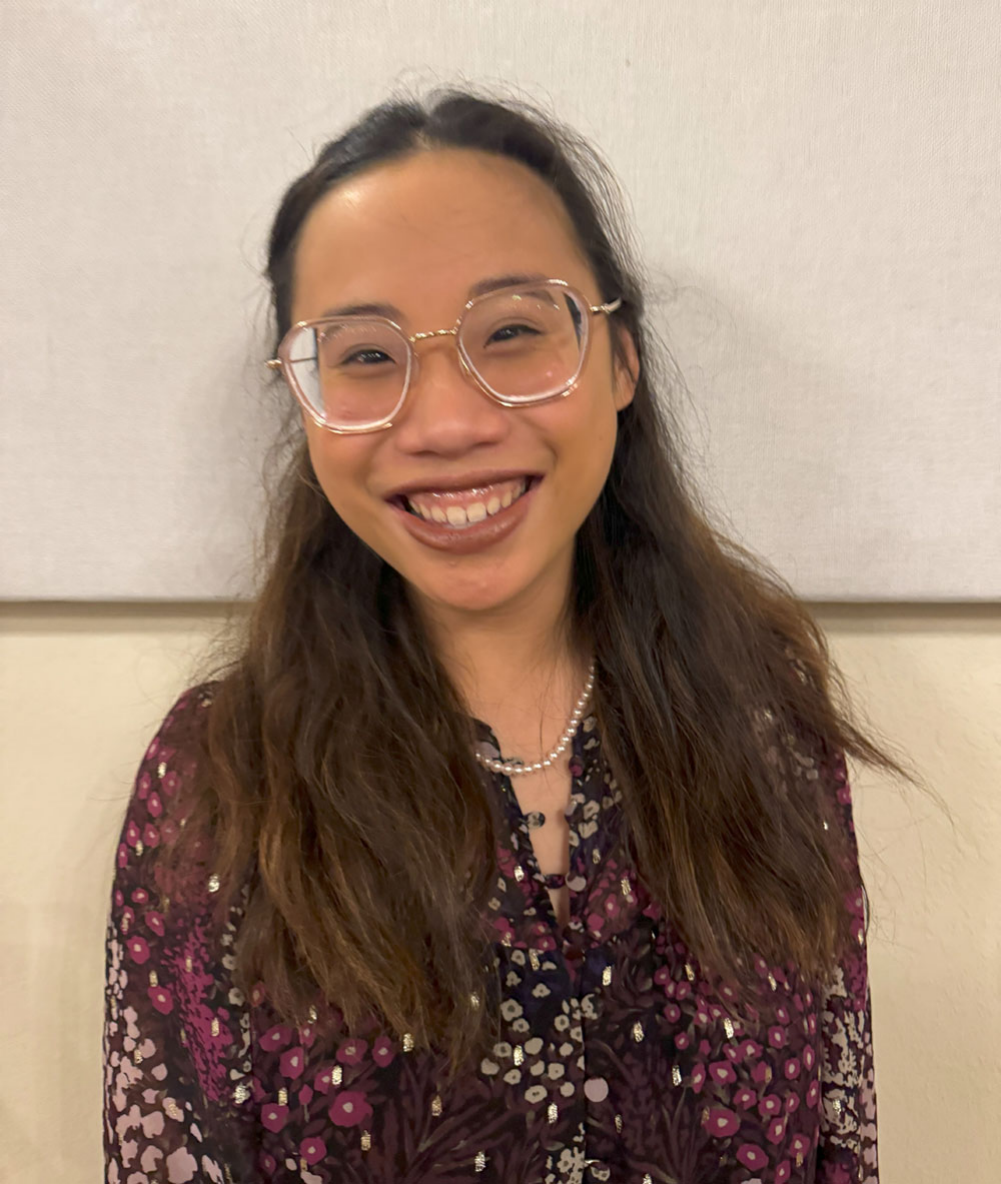


**Anna Lennon**

**Describe your scientific journey and your current research focus**

Initially studying education, my studies sparked a desire to ask deeper questions and become more actively involved in scientific research. Motivated by a love of science and desire to pursue the truth, my primary goal is to contribute to the body of scientific knowledge regarding the relationship between microbial cooperation and stress. The process of evolution shaping the modern world captured my attention in high school and has continued to fuel my curiosity and graduate work.

**Who or what inspired you to become a scientist?**

My middle school science teacher was a nun who was the first science teacher who displayed a deep love and passion for science. Her excitement around science stayed with me as I continued through school and fell deeper in love with science particularly biology.

We found that the fungus potentially secretes a neurotoxin that affects frogs' ability to respond to stimulus and escape predators

**How would you explain the main finding of your paper?**

Our study analysed how a fungal infection, *Batrachochytrium dendrobatidis*, influences the movement of frogs through the production and secretion of a neurotoxin. We found that the fungus potentially secretes a neurotoxin that affects frogs' ability to respond to stimulus and escape predators.

**What are the potential implications of this finding for your field of research?**

The delayed ability to escape threatens infected frog survivorship. While there are other direct mechanisms of mortality, the reduced locomotor performance is an indirect cause that still threatens amphibian populations.

**Figure:**
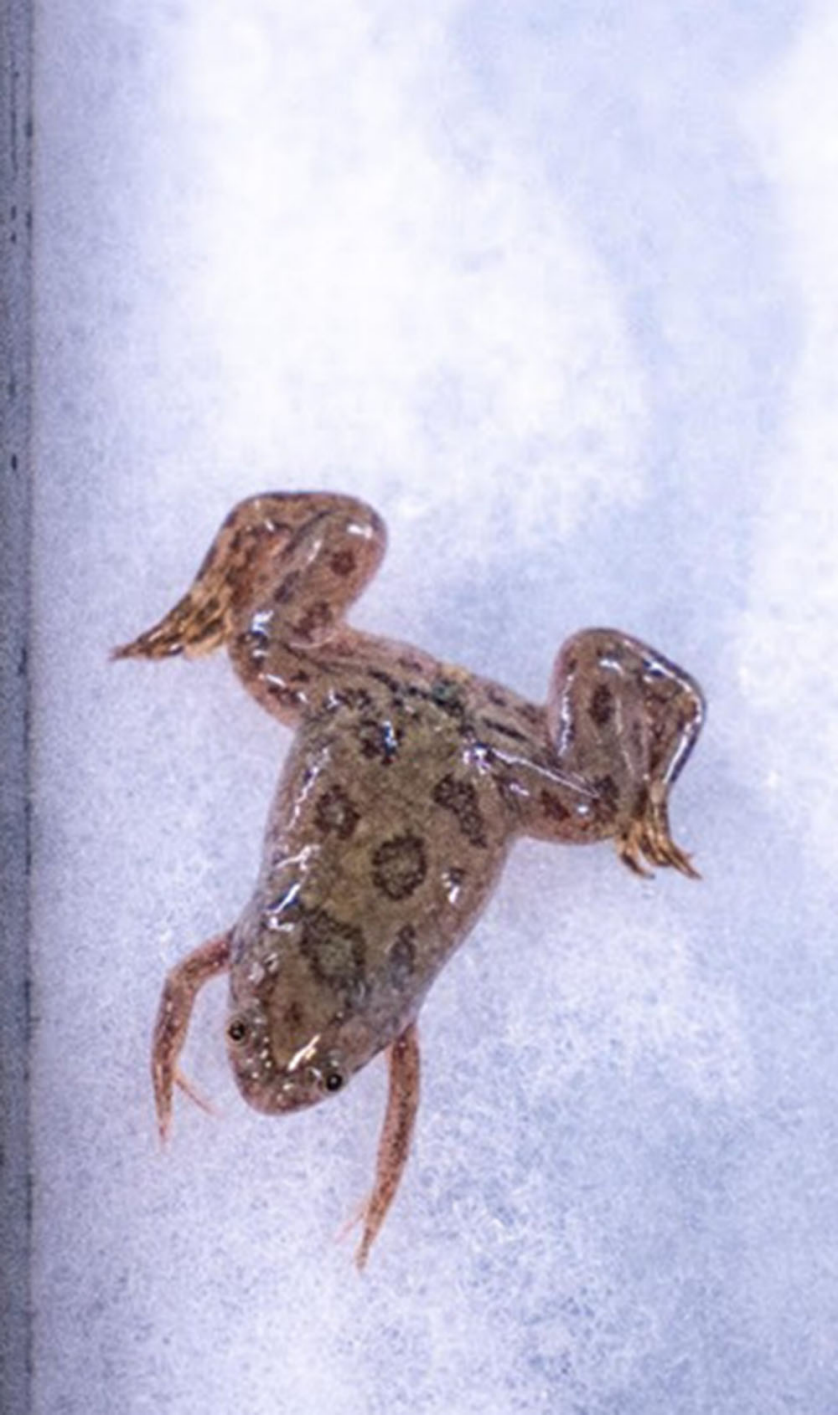
*Xenopus laevis.*

**Which part of this research project was the most rewarding?**

This project was incredibly rewarding as it was the first time I was the first author and able to claim full ownership of the project. From the initial idea to presenting the work at various conferences, I felt this idea and execution was completely mine under the mentorship of several faculty members at different institutions.

**What do you enjoy most about being an early-career researcher?**

The learning process and mentorship. As a graduate student, I am still in the process of formation and thus able to learn from many different individuals who have different approaches and ideas. I am then able to combine different training styles to better my research.

**What piece of advice would you give to the next generation of researchers?**

It is okay to ask for help. Often, we feel we must know everything, but you are early career and are not supposed to know everything. I started graduate school extremely young and as a result could approach my first year with greater curiosity and openness to mentorship.

**What's next for you?**

I am a second year PhD student in the Jay Lennon lab at Indiana University. My goal is to finish my degree and continue research at a federal institution in microbial evolution.
